# Mitochondrial ATP-Sensitive K+ Channel Opening Increased the Airway Smooth Muscle Cell Proliferation by Activating the PI3K/AKT Signaling Pathway in a Rat Model of Asthma

**DOI:** 10.1155/2021/8899878

**Published:** 2021-07-12

**Authors:** Min Gao, Qinran Sun, Qingfa Liu

**Affiliations:** ^1^Department of Respiratory, The First Affiliated Hospital of Shandong First Medical University, Jinan, Shandong, China; ^2^Department of Pain Management, The First Affiliated Hospital of Shandong First Medical University, Jinan, Shandong, China; ^3^Department of Respiratory Medicine, Liaocheng People's Hospital of Shandong Province, Liaocheng 252000, China

## Abstract

Abnormal proliferation of airway smooth muscle cells (ASMCs) leads to airway remodeling and the development of asthma. This study aimed to assess whether mitochondrial ATP-sensitive K+ (mitoK_ATP_) channels regulated the proliferation of ASMCs by regulating the phosphoinositide 3-kinase/protein kinase B (PI3K/AKT) pathway in asthmatic rats. Forty-eight Sprague Dawley rats were immunized with ovalbumin-containing alum to establish the asthma models. The ASMCs were isolated and identified by phase-contrast microscopic images and immunohistochemical staining for *α*-smooth muscle actin. The ASMCs were treated with a potent activator of mitoK_ATP_, diazoxide, or an inhibitor of mitoK_ATP_, 5-hydroxydecanoate (5-HD). Rhodamine-123 (R-123) was used for detecting the mitochondrial membrane potential (Δ*ψ*m). The proliferation of ASMCs was examined by the MTT (3-[4,5-dimethylthiazol-2-yl]-2,5 diphenyl tetrazolium bromide) assay. The protein and mRNA expressions of AKT and p-AKT were detected using western blotting and quantitative real-time PCR. The results showed that diazoxide enhanced the mitoK_ATP_ channel opening in ASMCs in the rat model of asthma, while 5-HD impeded it. Diazoxide also increased ASMC proliferation in the rat model of asthma, whereas 5-HD alleviated it. However, LY294002, a PI3K/AKT pathway inhibitor, reversed the functional roles of diazoxide in the proliferation ability of ASMCs in the rat model of asthma. Furthermore, treatment with diazoxide induced the phosphorylation of AKT, and treatment with 5-HD decreased the phosphorylation of AKT in ASMCs in the rat model of asthma. In conclusion, the mitoK_ATP_ channel opening increased the proliferation of ASMCs by activating the PI3K/AKT signaling pathway in a rat model of asthma.

## 1. Introduction

Asthma, characterized by airway remodeling, chronic airway inflammation, and airway hyperresponsiveness, posed a major threat to humans worldwide [1, 2]. Airway remodeling is mainly characterized by the epithelial-to-mesenchymal transition, mucous cell metaplasia, mucus hypersecretion, basement membrane thickening, collagen deposition, and hyperplasia of airway smooth muscle cells (ASMCs) [3–6]. When stimulated by extracellular factors, ASMCs perform a contractile function and undergo phenotypic transformation [7]. According to the previous research, ASMC proliferation is involved in airway remodeling and irreversible airway obstruction during severe asthma [8].

Mitochondrial ATP-sensitive potassium (mitoK_ATP_) channels are vital membrane proteins found in multiple cells and have been extensively studied in many types of cells, such as nerve cells, smooth muscle cells, cardiomyocytes, and skeletal muscle cells. Their cellular functions include bioelectricity activity and cellular energy metabolism [9, 10]. Previous research showed that mitoK_ATP_ channel activation strongly regulated the mitochondrial membrane potential (Δ*ψ*m) to prevent mitochondrial permeability transition and thus protect mitochondria during ischemia/reperfusion [11]. However, only a few studies have considered the role of mitoK_ATP_ channels in the proliferative ability of ASMCs in asthma. Recent studies indicated that the proliferative ability of human pulmonary arterial smooth muscle cells and ASMCs, in addition to phenotypic transformation and airway reconstruction, may be regulated by the opening of the mitoK_ATP_ channels and depolarization of Δ*ψ*m [12, 13]. However, the underlying mechanism remained unclear.

The phosphoinositide 3-kinase/protein kinase B (PI3K/AKT) pathway mediates various targets (e.g., glycogen synthase kinase-3, mammalian target of rapamycin, phosphodiesterase-3B, and insulin receptor substrate-1) to regulate cellular survival, growth, and proliferation through many mechanisms [14]. This signaling pathway plays a crucial role in inflammatory cell recruitment, regulating the expression and activation of inflammatory mediators, as well as in airway remodeling, immune cell function, and corticosteroid insensitivity in chronic inflammatory respiratory disease [14]. A previous study demonstrated that the PI3K/AKT pathway played a major role in the proliferation of ASMCs isolated from asthma patients [15].

This study aims to determine whether the mitoK_ATP_ channel plays a functional role in the proliferation ability of ASMC and whether this role is related to the PI3K/AKT signaling pathway, to provide a research basis for finding new targets for the treatment of asthma.

## 2. Materials and Method

### 2.1. Generation of the Asthma Rat Model

This study used 48 male Sprague Dawley (SD) rats (weight: 250–350 g) maintained under a 12 h light/dark cycle in a temperature- (22 ± 2°C) and humidity- (45–60%) controlled environment. The animals had access to food and water ad libitum. The rats were equally divided into the asthma group and the control group. The rats in the asthma group were intraperitoneally injected with 10% ovalbumin (OVA) plus alum on days 0 and 14, whereas the control rats received normal saline [16]. The rats in the asthma group were then challenged with OVA aerosols (1% in PBS; 30 min/d) for 7 consecutive days. As a control, rats in the control group were sensitized with saline. The experiments were approved by the ethics committee of our hospital and conducted according to the guidelines for the care and use of animals.

### 2.2. ASMC Preparation and Culture

Primary ASMCs were isolated from the tissues of the asthmatic and control rats. Smooth muscle was separated from parenchyma and connective tissue by tissue digestion with the solution consisting of 1 mg/mL of collagenase I, 10 mg/mL of BSA, and 1 mg/mL of papain in D-Hanks solution at 37°C for 15 min. The separated cells were then cultured in Dulbecco's modified Eagle's medium (DMEM) containing 20% FBS and seeded in cultured flasks with 10% FBS-DMEM. They were passaged when they reached 80–90% confluence [17]. The ASMCs at the 3rd to 6th generations were used for performing the experiments. The primary ASMCs obtained from the asthma rat model were grouped as follows: (i) asthma group; (ii) asthma + diazoxide group, which received an additional treatment of diazoxide (a selective mitoK_ATP_ channel opener) (diluted in DMSO; 100 *μ*mol/L); and (iii) asthma + 5-HD group, which received an additional treatment of 5-HD (5-hydroxydecanoate, a selective mitoK_ATP_ channel blocker) (diluted in DMSO; 500 *μ*mol/L).

The ASMCs obtained from the normal rats were also divided into 3 groups as follows: normal group, normal + diazoxide group, and normal +  5-HD group, as described above.

### 2.3. Identification of ASMCs

Phase-contrast microscopic images were taken to identify the specific morphological features of the ASMCs. The ASMCs were identified by immunohistochemical staining for *α*-smooth muscle actin.

### 2.4. Measurement of Rhodamine Fluorescence

The ASMCs were cultured and grown in six-well plates loaded with rhodamine-123 (R-123; 10 *μ*g/mL) and incubated for 30 min at 37°C in the dark. R-123 fluorescence was quenched at resting Δ*ψ*m and increased when the mitochondrial membrane was depolarized and was selectively taken up by mitochondria, thus revealing a linear correlation between R-123 fluorescence and Δ*ψ*m. After treatment with diazoxide or 5-HD, unabsorbed R-123 in the ASMCs was washed out by D-Hanks solution to ensure that it did not affect the fluorescence intensity. The fluorescence was stimulated at 488 nm and detected at 530 nm by laser confocal microscopy. The results were analyzed using an HPIAS-1000 image analyzer.

### 2.5. MTT Assay

The ASMCs were cultured in 96-well plates at a cell density of 1 × 10^4^ cells/mL at 37°C and 5% CO_2_, in a culture medium, which was replaced with the fresh medium every 2 days. After adherent cell cultures were established, the ASMCs were treated with diazoxide or 5-HD as previously described. The medium was aspirated from the test wells, and PBS was used to gently wash the cells. MTT solution was added and incubated for 4 h, followed by the addition of DMSO (1.5 mL). Then, the supernatant was carefully aspirated. Absorbance was measured directly by spectrophotometry at 570 nm. LY294002 (a PI3K/AKT pathway inhibitor) with a final concentration of 25 mmol/L was added into the cell culture medium in the asthma group and asthma + diazoxide group, and the absorbance value was measured.

### 2.6. Western Blotting

After 2 days, the cells were washed with PBS and lysed in lysis buffer containing 0.5 M Tris-HCl (pH 7.4), 1.5 M NaCl, 2.5% deoxycholic acid, 10% NP-40, 10 mM EDTA, and protease inhibitors (Millipore, Bedford, MA) and subsequently incubated on ice for 30 min. The mixtures were then centrifuged (13,000 rpm; 30 min). The protein concentrations were measured with a BCA kit. The protein extracts were electrophoresed on 6% Bis-Tris protein gels and then transferred to PVDF membranes. After incubated in 5% dry milk for 1 h, the membranes were incubated with the following primary antibodies: anti-p-AKT, anti-AKT (BD Biosciences), and glyceraldehyde-3-phosphate dehydrogenase (GAPDH) (Bioworld, USA). Then, the membranes were incubated with the peroxidase AffiniPure goat anti-rabbit IgG (H + L chains; Jackson ImmunoResearch Laboratories, West Grove, PA). The enhanced chemiluminescence (ECL) kit (Amersham, UK) was used to detect the protein bands. The Image-Pro Plus image analysis software was used to quantify the band intensity. The mean grey value was used for further statistical analysis.

### 2.7. Reverse-Transcription PCR and Quantitative PCR

Total RNA was extracted from the ASMCs using TRIzol (Invitrogen, Carlsbad, CA, USA) according to the manufacturer's instructions. Then, the RNA was mixed with the oligo (dT) primers and reverse-transcribed to cDNA using a First-Strand cDNA Synthesis Kit (Fermentas, Burlington, Canada). Afterward, the qPCR was performed using an SYBR premix Ex Taq kit (TaKaRa, Dalian, China) in an ABI 7900 Imaging System (UVP, LLC Upland, CA, USA). The PCR conditions were as follows: 94°C for 30 seconds followed by 40 cycles of 95°C for 5 seconds, 56°C for 30 seconds, and 72°C for 60 seconds. Then, the PCR products were separated on 2% agarose gels. All the reactions were performed in triplicate and the levels of gene expression were calculated by the relative quantification method. The GAPDH was used as an endogenous reference gene. All the primers (Takara Biotechnology Co. Ltd., Dalian, China) are listed in Table 1.

### 2.8. Statistical Analysis

All the data are shown as mean ± standard deviation and analyzed by the SPSS statistical software (version 20.0). The *t*-test and one-way analysis of variance (ANOVA) test were used for comparisons between two groups or among multiple groups, respectively. *p* < 0.05 was considered statistically significant.

## 3. Results

### 3.1. Characterization and Identification of ASMCs

**Figure 1:**
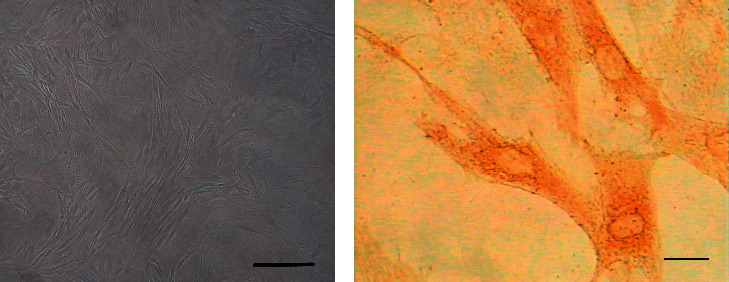
Identification of airway smooth muscle cells (ASMCs) by morphological characteristics and immunohistochemical staining. (a) ASMCs displayed the characteristic “hill and valley” appearance (phase-contrast microscope, ×100). (b) Positive expression for *α*-actin in ASMCs (immunocytochemical staining, ×400).

The ASMCs were identified according to the specific morphological characteristic (i.e., “hill and valley” feature) of these cells using phase-contrast microscopy. Immunohistochemical staining for *α*-smooth muscle actin was also performed to identify ASMCs. The cultured cells were found to be positive for *α*-smooth muscle actin. These experiments demonstrated the identification of ASMCs (Figure 1).

### 3.2. Δ*ψ*m in ASMCs in the Asthma Rat Model

**Figure 2:**
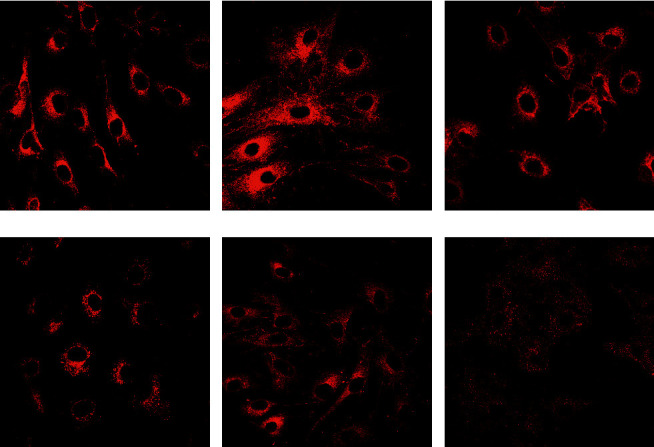
The mitochondrial membrane potential Δ*ψ*m in airway smooth muscle cells (ASMCs) in the asthma rat model. (a) Asthma group; (b) asthma + diazoxide group; (c) asthma + 5-HD group; (d) normal group; (e) normal + diazoxide group; (f) normal + 5-HD group; 5-HD, 5-hydroxydecanoate.

Δ*ψ*m in ASMCs was detected using rhodamine fluorescence staining. In the asthma group (Figure 2(a)), the fluorescence intensity was markedly higher than that in the normal group (Figure 2(d)). Diazoxide significantly increased the fluorescence intensity of R-123 in the normal + diazoxide (Figure 2(e)) group as compared with that in the normal group (Figure 2(d)). The fluorescence intensity in the asthma + diazoxide group (Figure 2(b)) was also strikingly higher than that in the asthma group (Figure 2(a)). Conversely, the fluorescence intensity of R-123 in the asthma + 5-HD group (Figure 2(c)) was lower than that in the asthma group (Figure 2(a)). The Δ*ψ*m is mainly produced when there is a proton gradient and the protons pass through the mitochondrial inner membrane. These results indicated that diazoxide enhanced the mitoK_ATP_ channel opening in ASMCs in the asthma rat model, whereas 5-HD impeded the channel opening.

### 3.3. Effect of MitoK_ATP_ Channel Opening on the Proliferation of ASMCs from the Asthma Rat Model

**Figure 3:**
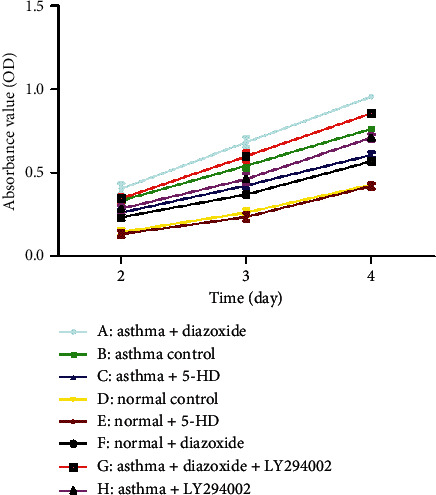
Effect of the mitochondrial ATP-sensitive potassium (mitoK_ATP_) channel opening on the proliferation of airway smooth muscle cells (ASMCs) in the asthma rat model. The proliferation of ASMCs in the different groups was measured by the MTT assay. 5-HD, 5-hydroxydecanoate.

The MTT method was used to detect the proliferation of ASMCs. As shown in Figure 3, the absorbance values of ASMCs in the normal + diazoxide group and asthma control group were markedly higher than those in the normal group (*p* < 0.05). The absorbance value in the asthma + diazoxide group was also significantly higher than that in the asthma group (*p* < 0.05). However, the absorbance value of ASMCs in the asthma + 5-HD group was lower than that in the asthma group (*p* < 0.05). There was no significant difference in the absorbance value between the normal control group and the normal + 5-HD group (*p* > 0.05). These results suggested that diazoxide increased the proliferation of ASMCs in the asthma rat model, and 5-HD decreased it.

### 3.4. Effect of MitoK_ATP_ Channel Opening on the PI3K/AKT Signaling Pathway in ASMCs in the Asthma Rat Model

**Figure 4:**
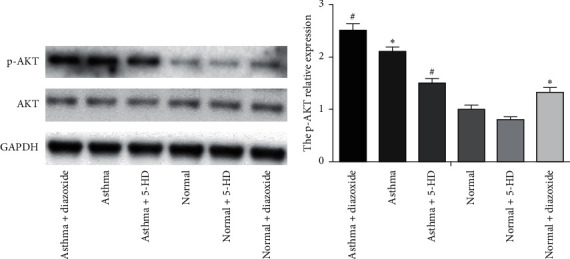
The protein expression of protein kinase B (AKT) and p-AKT in airway smooth muscle cells (ASMCs) by the western blot analysis. (a) Representative protein expression profiles. (b) The results shown in (a) were quantified and graphed by densitometry using Image-Pro Plus software. The results were expressed as the mean ± SD. ^*∗*^*p* < 0.05 versus the normal group, ^*#*^*p* < 0.05 versus the asthma group. GAPDH: glyceraldehyde-3-phosphate dehydrogenase; 5-HD, 5 hydroxydecanoate.

As shown in Figure 3, the absorbance value of the asthma + LY294002 group was lower than that of the asthma group (*p* < 0.05) and the absorbance value of the asthma + diazoxide + LY294002 group was also lower than that of the asthma + diazoxide group (*p* < 0.05), indicating that LY294002 (a PI3K/AKT pathway inhibitor) reversed the effects of diazoxide on the proliferative ability of ASMCs in the rat model of asthma. In addition, the expression levels of AKT and pAKT in the ASMCs treated with diazoxide and 5-HD in the normal and asthma groups were detected by Western blot analysis. There was no significant difference in the protein expression levels of AKT in the different groups (Figure 4). However, as compared with the normal group, the expression levels of the p-AKT protein were higher in the normal + diazoxide group and asthma group (*p* < 0.05). Furthermore, the p-AKT protein level was higher in the asthma + diazoxide group and lower in the asthma + 5-HD group as compared with that in the asthma group (*p* < 0.05) (Figure 4). The results of AKT mRNA expression detected by the real-time quantitative PCR were consistent with those of the western blot (Table 2). Therefore, the treatment with diazoxide induced AKT phosphorylation, and the treatment with 5-HD decreased AKT phosphorylation in ASMCs in the rat model of asthma. These results suggested that mitoK_ATP_ channel opening modulated the PI3K/AKT signaling pathway in asthmatic rats.

## 4. Discussion

Asthma is a chronic disease associated with hyperresponsiveness, obstruction, and remodeling of the airways. Structural changes are related to airway remodeling, including epithelial cell shedding, goblet cell metaplasia or hyperplasia, subepithelial fibrosis, bronchial neovascularization [18], and smooth muscle cell hyperplasia [19]. ASMCs are effector cells of bronchoconstriction and produce inflammatory mediators and angiogenic factors. Increased ASMC levels may cause airway remodeling and persistent airflow limitation [20]. Previous research showed that ASMCs were strongly associated with the pathogenesis of asthma [21].

MitoK_ATP_ channels play vital roles in mitochondrial ion balance in various cells and are involved in various cellular functions [22]. MitoK_ATP_ proteins maintain the potassium balance in mitochondria, and opening and closing of mitoK_ATP_ channels can change Δ*ψ*m. In the present study, diazoxide was used to selectively open and activate mitoK_ATP_ channels, and 5-HD was used as a selective blocker to specifically diminish mitoK_ATP_ channel activity [23]. The Δ*ψ*m in ASMCs in the different groups was investigated using R-123 fluorescence staining. The results shown in Figure 2 indicated that the diazoxide treatment resulted in mitoK_ATP_ channel opening in ASMCs. Conversely, 5-HD significantly reduced mitoK_ATP_ channel opening, as evidenced by the decreased fluorescence intensity of R-123.

Previous research demonstrated that mitoK_ATP_ channel activation resulted in ASMC proliferation and secretion in asthmatic rats [24]. In this study, we examined the effects of mitoK_ATP_ channel opening on the proliferative ability of ASMCs using an MTT assay. According to the MTT assay results, the AMSC proliferation increased in the bronchial asthmatic rats challenged with OVA as compared with that in the normal controls (Figure 3). Furthermore, the absorbance values observed in the asthma + diazoxide group were significantly higher than those in the asthma group, whereas the absorbance values in the asthma + 5-HD group were much lower than those in the asthma group. The results suggested that diazoxide increased ASMC proliferation in the asthma rat model, and 5-HD decreased it. These results pointed to mitoK_ATP_ channel opening leading to enhanced ASMC proliferation. These findings were consistent with previously reported results [25]. A recent study indicated that depolarization of the Δ*ψ*m and mitoK_ATP_ channel opening might result in the proliferation of human pulmonary arterial smooth muscle cells [12]. Another study reported that depolarization of the Δ*ψ*m and mitoK_ATP_ channel opening contributed to the cell proliferative and apoptotic abilities of ASMCs induced by asthma [13]. 

PI3K/AKT is closely associated with various functions in cell proliferation, cell survival, and cancer progression [26, 27]. AKT, a major downstream target of PI3K, is activated by various stimuli, hormones, and growth factors. AKT phosphorylation can be used as an index of PIK3 activity. PI3Ks are a family of proteins involved in cell proliferation, differentiation, apoptosis, and glucose transportation. Proliferation is influenced by several serine/threonine kinase cascades and many cellular signaling pathways, including PI3K/AKT signaling pathways. A previous study showed that inhibition of PI3K/AKT signaling may attenuate ASMC proliferation [28]. Recently, Dai et al. provided evidence that inhibition of TGF-*β*1-induced ASMC proliferation was associated with the PI3K/AKT inactivation [29]. However, the interactions between the mitoK_ATP_-dependent proliferation of ASMCs and the PI3K/AKT signaling pathway remained uncertain. In the present study, LY294002, a PI3K/AKT pathway inhibitor, reversed the effects of diazoxide on the proliferative ability of ASMCs in the asthma rat model, indicating that the PI3K/AKT pathway may be involved in the proliferative ability of ASMCs and the progression of asthma. Our results also indicated that diazoxide treatment induced AKT phosphorylation and that 5-HD treatment decreased AKT phosphorylation in ASMCs in an asthma rat model. These results suggested that the phosphorylation of AKT relies on the mitoK_ATP_ channel opening. Taken together, it can be concluded that, in ASMCs, the mitoK_ATP_ channel opening activates the PI3K/AKT signaling pathway, thereby increasing cell proliferation and aggravating airway remodeling.

## 5. Conclusions

The findings of this study provide the evidence that in ASMCs under anaerobic conditions, asthma may lead to activation and opening of mitoK_ATP_ channels and partial depolarization of the ∆*ψ*m, which further promotes the cell proliferation of ASMCs by activating the PI3K/AKT signaling pathway. These findings reveal that mitoK_ATP_ channel opening and the PI3K/AKT signaling pathway play important roles in the pathogenesis of asthma. Furthermore, they suggest that mitoK_ATP_ channels may represent a new therapeutic target in the treatment of asthma.

## Figures and Tables

**Table 1:** The PCR primer sequences.

Gene	Sequence	Size (bp)	Tm (°C)
AKT			
Sense	ATGGACTTCCGGTCAGGTTCA	126	62
Antisense	GCCCTTGCCCAGTAGCTTCA		
GAPDH			
Sense	GGCACAGTCAAGGCTGAGAATG	143	61.5
Antisense	ATGGTGGTGAAGACGCCAGTA		

*Note.* All sequences are shown in the 5′ to 3′ orientation. bp: base pair; Tm: temperature; AKT: protein kinase B; GAPDH: glyceraldehyde-3-phosphate dehydrogenase.

**Table 2:** The mRNA expression of protein kinase B (AKT) in airway smooth muscle cells (ASMCs) in the asthma rat model.

Group	*N*	GAPDH-Ct (Ct-G)	AKT-Ct (Ct-AKT)	2^−ΔΔCt^
Normal group	9	17.87 ± 0.64	25.01 ± 0.60	1.00
Normal + diazoxide group	9	19.66 ± 0.27	26.66 ± 0.16	1.14 ± 0.26
Normal + 5-HD group	9	19.32 ± 1.05	27.26 ± 0.56	0.98 ± 0.26
Asthma group	9	18.36 ± 0.26	25.49 ± 0.48	0.97 ± 0.32
Asthma + diazoxide group	9	15.10 ± 0.53	22.11 ± 0.43	1.12 ± 0.30
Asthma + 5-HD group	9	16.66 ± 0.57	23.66 ± 0.55	1.13 ± 0.27

The mRNA expression levels of the AKT in ASMCs were determined by comparative Ct value, and the results were expressed as the mean ± SD.

## Data Availability

The analyzed datasets generated during the study are available from the corresponding author upon reasonable request.

## References

[1] Nakagome K., Nagata M. (2011). Pathogenesis of airway inflammation in bronchial asthma. *Auris Nasus Larynx*.

[2] Holgate S. T. (2008). Pathogenesis of asthma. *Clinical & Experimental Allergy*.

[3] Bai T. R. (2010). Evidence for airway remodeling in chronic asthma. *Current Opinion in Allergy & Clinical Immunology*.

[4] Bergeron C., Al-Ramli W., Hamid Q. (2009). Remodeling in asthma. *Proceedings of the American Thoracic Society*.

[5] Fahy J. V., Corry D. B., Boushey H. A. (2000). Airway inflammation and remodeling in asthma. *Current Opinion in Pulmonary Medicine*.

[6] Halwani R., Al-Muhsen S., Hamid Q. (2010). Airway remodeling in asthma. *Current Opinion in Pharmacology*.

[7] Sukkar M. B., Stanley A. J., Blake A. E. (2004). ‘Proliferative’ and ‘synthetic’ airway smooth muscle cells are overlapping populations. *Immunology & Cell Biology*.

[8] Yeganeh B., Xia C., Movassagh H. (2013). Emerging mediators of airway smooth muscle dysfunction in asthma. *Pulmonary Pharmacology & Therapeutics*.

[9] Teshima Y., Akao M., Li R. A. (2003). Mitochondrial ATP-sensitive potassium channel activation protects cerebellar granule neurons from apoptosis induced by oxidative stress. *Stroke*.

[10] Yamauchi T., Kashii S., Yasuyoshi H., Zhang S., Honda Y., Akaike A. (2003). Mitochondrial ATP-sensitive potassium channel: a novel site for neuroprotection. *Investigative Opthalmology & Visual Science*.

[11] Korge P., Honda H. M., Weiss J. N. (2002). Protection of cardiac mitochondria by diazoxide and protein kinase C: implications for ischemic preconditioning. *Proceedings of the National Academy of Sciences*.

[12] Hu H., Wang T., Zhang Z., Zhao J., Xu Y. (2006). The effect of mitochondrial membrane potential on changes of reactive oxygen species and on proliferation of hypoxic human pulmonary arterial smooth muscle cells. *Chinese Journal of Tuberculosis and Respiratory Diseases*.

[13] Zhao J.-P., Gao M., Ye Y.-J., Hu W.-H., Zhou Z.-G., Hu H.-L. (2009). Regulation of rat airway smooth muscle cell proliferation by mitochondrial ATP-sensitive K (+) channel in asthmic rats. *Sheng Li Xue bao:[Acta Physiologica Sinica]*.

[14] Hofler A., Nichols T., Grant S. (2011). Study of the PDK1/AKT signaling pathway using selective PDK1 inhibitors, HCS, and enhanced biochemical assays. *Analytical Biochemistry*.

[15] Burgess J. K., Lee J. H., Ge Q. (2008). Dual ERK and phosphatidylinositol 3‐kinase pathways control airway smooth muscle proliferation: differences in asthma. *Journal of Cellular Physiology*.

[16] Salmon M., Walsh D. a., Koto H., Barnes P. j., Chung K. f. (1999). Repeated allergen exposure of sensitized Brown-Norway rats induces airway cell DNA synthesis and remodelling. *European Respiratory Journal*.

[17] Johnson P. R. A., Roth M., Tamm M. (2001). Airway smooth muscle cell proliferation is increased in asthma. *American Journal of Respiratory and Critical Care Medicine*.

[18] Shifren A., Witt C., Christie C., Castro M. (2012). Mechanisms of remodeling in asthmatic airways. *Journal of Allergy*.

[19] Berair R., Hollins F., Brightling C. (2013). Airway smooth muscle hypercontractility in asthma. *Journal of Allergy*.

[20] Al-Muhsen S., Johnson J. R., Hamid Q. (2011). Remodeling in asthma. *Journal of Allergy and Clinical Immunology*.

[21] Oenema T. A., Smit M., Smedinga L. (2012). Muscarinic receptor stimulation augments TGF-*β*1-induced contractile protein expression by airway smooth muscle cells. *American Journal of Physiology-Lung Cellular and Molecular Physiology*.

[22] Garlid K. D., Dos Santos P., Xie Z.-J., Costa A. D., Paucek P. (2003). Mitochondrial potassium transport: the role of the mitochondrial ATP-sensitive K+ channel in cardiac function and cardioprotection. *Biochimica et Biophysica Acta (BBA)-Bioenergetics*.

[23] O’Rourke B. (2000). Myocardial KATP channels in preconditioning. *Circulation Research*.

[24] Chen C., Wang R., Zhou S., Zhao J., Xu Y. (2014). Effects of mitochondrial ATP-sensitive potassium channels on the proliferation and secretion of human airway smooth muscle cells. *Iranian Journal of Allergy, Asthma and Immunology*.

[25] Wan X., Zhao J., Xie J. (2012). Effects of mitochondrial ATP-sensitive K+ channel on protein kinase C pathway and airway smooth muscle cell proliferation in asthma. *Journal of Huazhong University of Science and Technology (Medical Sciences)*.

[26] Coffer P. J., Jin J., Woodgett J. R. (1998). Protein kinase B (c-Akt): a multifunctional mediator of phosphatidylinositol 3-kinase activation. *Biochemical Journal*.

[27] Kandel E. S., Hay N. (1999). The regulation and activities of the multifunctional serine/threonine kinase Akt/PKB. *Experimental Cell Research*.

[28] Stewart A. G., Xia Y. C., Harris T., Royce S., Hamilton J. A., Schuliga M. (2013). Plasminogen-stimulated airway smooth muscle cell proliferation is mediated by urokinase and annexin A2, involving plasmin-activated cell signalling. *British Journal of Pharmacology*.

[29] Dai Y., Li F., Wu L. (2014). Roxithromycin treatment inhibits TGF-*β*1-induced activation of ERK and AKT and down-regulation of caveolin-1 in rat airway smooth muscle cells. *Respiratory Research*.

